# A Novel Fault Diagnosis Approach for Chillers Based on 1-D Convolutional Neural Network and Gated Recurrent Unit

**DOI:** 10.3390/s20092458

**Published:** 2020-04-26

**Authors:** Zhuozheng Wang, Yingjie Dong, Wei Liu, Zhuo Ma

**Affiliations:** Faculty of Information Technology, Beijing University of Technology, Beijing 100124, China; yingjied@emails.bjut.edu.cn (Y.D.); liuwei0823@bjut.edu.cn (W.L.); mazhuo927@163.com (Z.M.)

**Keywords:** fault diagnosis, chiller system, one-dimensional convolutional neural network, time-series sequences, gated recurrent unit

## Abstract

The safety of an Internet Data Center (IDC) is directly determined by the reliability and stability of its chiller system. Thus, combined with deep learning technology, an innovative hybrid fault diagnosis approach (1D-CNN_GRU) based on the time-series sequences is proposed in this study for the chiller system using 1-Dimensional Convolutional Neural Network (1D-CNN) and Gated Recurrent Unit (GRU). Firstly, 1D-CNN is applied to automatically extract the local abstract features of the sensor sequence data. Secondly, GRU with long and short term memory characteristics is applied to capture the global features, as well as the dynamic information of the sequence. Moreover, batch normalization and dropout are introduced to accelerate network training and address the overfitting issue. The effectiveness and reliability of the proposed hybrid algorithm are assessed on the RP-1043 dataset; based on the experimental results, 1D-CNN_GRU displays the best performance compared with the other state-of-the-art algorithms. Further, the experimental results reveal that 1D-CNN_GRU has a superior identification rate for minor faults.

## 1. Introduction

**Figure 1:**
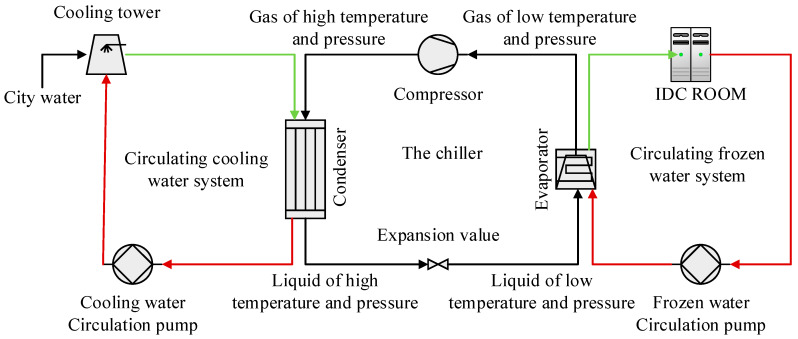
The refrigeration system of IDC.

With the rapid development of computer and sensor technology, modern industrial systems present a tendency towards complexity and integration, and the data reflecting the operation mechanism and state of the system presents all the characteristics of “big data”. As the most vital equipment of the refrigeration system in an Internet Data Center (IDC), the chiller is primarily composed of the condenser, the evaporator, the compressor, the expansion valve, the cooling water circulation system, as well as the chilling water circulation system, as illustrated in Figure 1. The primary function of the chiller is to provide cooling source for the IDC room and guarantee the normal operation of the data center. The chiller fault occurrence not only shortens the equipment service life, decreasing the system performance, but also results in the loss of the information stored in the servers, bringing severe and even irreversible economic losses [1]. The accurate fault diagnosis, therefore, is of great significance to the safety of the IDC.

The commonly used methods for fault diagnosis can be divided into three categories: model-based fault diagnosis, knowledge-based fault diagnosis and data-driven fault diagnosis [2]. Model-based fault diagnosis methods are based on the internal mechanism of system, which estimates the system by constructing the mathematical model that is sensitive to specific faults and achieves fault diagnosis through the deviation between estimates and measurements [3]. However, this method is not scalable, and each model can only be used for each specific system. 

**Figure 2:**
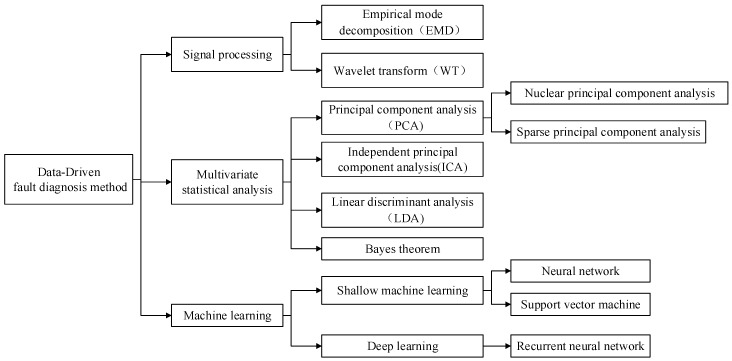
The data-driven fault diagnosis method for chillers.

Knowledge-based fault diagnosis methods do not depend on mathematical or physical models, but rather the diagnosis results are determined by the expert experience and the level of expert knowledge [4]. The data-driven fault diagnosis methods mainly use various data mining techniques to extract historical data features during the operation of the equipment and realize fault diagnosis by judging the consistency of the current data and those historical data features. Among them, the data-driven fault diagnosis method has often been used for fault diagnosis of chillers in recent years, including multivariate statistical analysis methods, signal processing methods, and machine learning methods, as shown in Figure 2.

Support Vector Machine (SVM) [5], Back Propagation Neural Network (BPNN) [6], multivariate statistical analysis methods comprising PCA [7] or ICA [8], are also called traditional intelligent fault diagnosis methods. References [9,10] studied the fault detection and diagnosis of chiller sensors and established a PCA-based diagnostic model. Compared with reference [9], reference [10] introduced a wavelet analysis method on the basis of PCA, effectively filtering the noise in the sensor fault information and improved the deficiencies of the principal component method in the fault detection and diagnosis of chiller sensors. As we all know, the main component of PCA is a linear combination of various variables. When the values of some variables are similar, this can lead to poor sensitivity. To solve the problem of insufficient sensitivity of the PCA method, reference [11] proposed a fault diagnosis method for air-conditioning sensors based on sparse principal components. This method used an elastic net to sparse the load matrix, reduce the association between principal components and variables, enhance the interpretability of principal components, and thereby improved the sensitivity of fault detection. In addition, reference [8] used the independent component analysis (ICA) method to extract the correlation of the chiller variables and reduce the dimensionality of the measurement data. This method checked whether the chiller fails by counting and calculating the threshold of the statistic. Experimental results showed that the method is very sensitive to early failures and can effectively reduce the rate of false negatives and false positives. This method improves the diagnostic performance of the PCA model to a certain extent. In order to solve the generalization ability of the fault diagnosis algorithm on the chiller multi-classification problem, reference [12] used the support vector machine (SVM) method to classify seven common chiller faults. Reference [13] established a BP-based neural network diagnostic model for typical local faults and system faults of centrifugal chiller units. By adjusting the network structure and parameters, and changing the training function to optimize the model, a good diagnosis effect was obtained in the local fault diagnosis of the chiller. However, due to the widespread impact of system failures (such as refrigerant leakage) on refrigeration systems, it is difficult to identify. Against the defects of error back-propagation neural network in chiller fault diagnosis, reference [14] used particle swarm optimization (PSO) to apply the optimized weights and thresholds model to the fault diagnosis of centrifugal chiller. The experimental results showed that compared with the traditional BP neural network, the optimized BP by PSO has significantly improved fault diagnosis performance, and the false alarm rate of fault diagnosis is reduced, and system faults, especially refrigerant leakage faults, are significantly improved. 

However, the chiller is a highly non-linear complex system. These traditional intelligent fault diagnosis methods face difficulties in representing complex functions and take a lot of time to extract effective features due to their poor performance and generalization ability. Moreover, they perform feature extraction and diagnosis separately, which will affect the final diagnosis performance.

Compared with the traditional intelligent fault diagnosis methods, deep learning methods contain a multi-layer hidden structure that may realize the feature matrix transformation layer by layer and guarantee effective feature extraction adaptively [15,16]. Deep learning, on the other hand, can approach complex functions better; thus, it may deal with high-dimensional and non-linear data efficiently and avoid the issue of insufficient diagnostic capability through multiple non-linear transformations and approximate complex non-linear functions [17]. Although fault diagnosis based on deep learning has attracted extensive attention in industry and academia, there are relatively few studies on fault diagnosis of chillers. Reference [18] offered a method on the basis of LSTM to diagnosis the fault of chillers, and obtained outstanding performance. Applying LSTM, reference [19] offered a fault detection and diagnosis method for the sensors of an air conditioning system. The fixed and drifting biases of both liquid line and discharge temperature sensors were successfully identified by building the fault detection and diagnosis models, respectively.

In this study, the collected data set from a chiller contains time-series data of multi-sensors and presents the “big data” characteristic. In deep learning, RNN and 1D-CNN are more capable of capturing connections in the time dimension. During the RNN variations, GRU improves the training efficiency by simplifying connection and reducing trainable parameters on the premise of ensuring the memory ability of neurons compared to LSTM. Therefore, a new approach for feature extraction and fault diagnosis is constructed based on 1D-CNN and GRU for the chiller fault diagnosis in this paper. Experiences evaluate the feasibility of the proposed diagnosis model on datasets with four levels of severity. Besides, the advantages of the model are verified by comparing it with 1D-CNN, GRU, LSTM, BPNN, PCA_BPNN, as well as 1D-CNN_LSTM. The leading contributions of this study may be summarized as below:

(1) Applying 1D-CNN and GRU, an innovative approach for feature extraction and fault diagnosis of the chiller is introduced in this article. The proposed approach can implement automatically features extraction from raw sensor data and fault diagnosis, simultaneously [3];
(2) The experiments are performed on 4 kinds of datasets with different fault severity; the experimental results reveal that the proposed fault diagnosis algorithm has a reasonable identification rate for minor faults.

The remainder of this paper is structured as follows: In Section 2, the experimental platform is introduced and the experimental data are briefly analyzed. The preparations related to the proposed approach and detailed descriptions of the proposed approach are presented in Section 3. The diagnosis process and evaluation are presented in Section 4. The experimental results and corresponding analysis are offered in Section 5, respectively. To end, the conclusions are provided in Section 6.

## 2. Experimental Platform and Data Analysis

The ASHRAE RP-1043 dataset is introduced in this paper in order to validate the effectiveness of the proposed approach. It belongs to a project sponsored by the American Society of Heating Refrigerating and Air-Conditioning Engineers (ASHRAE) in the 1990s to study faults in chillers [20].

### 2.1. Research Object

**Figure 3:**
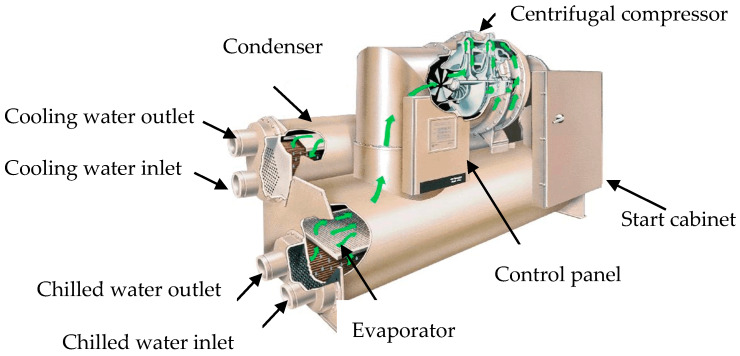
Structure of the centrifugal chiller.

The experimental object is a 316 kW centrifugal chiller, as shown in Figure 3. Seven typical faults were simulated through experiments, as provided in Table 1: (1) Reduced condenser water flow and reduced evaporator water flow—these faults can be simulated by adjusting the head pressure across the water pump; (2) Refrigerant leak and refrigerant overcharge—these faults can be simulated by removing or adding refrigerant from the system; (3) Excess oil—this fault can be simulated by adding oil to the system; (4) Condenser fouling—this fault can be simulated by plugging tubes in the condenser; (5) Non-condensable in the refrigerant—this fault can be simulated by adding nitrogen to the system.

For each single fault, four fault levels are simulated, with the severity increasing from level 1 to level 4, as shown in Table 2.

### 2.2. Experimental Platform

**Figure 4:**
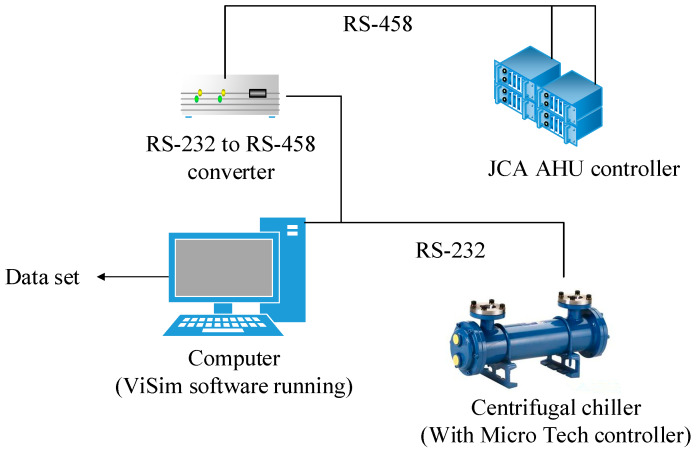
The data acquisition system.

The experimental platform obtains status operation data by equipping it with a large number of sensors on the evaporator side and condenser side of the chiller, mainly including temperature sensors, pressure sensors, flow sensors, and position sensors [21], which communicate with the PC through the RS-232 serial port, and a control processing unit (JCI AHU) is connected to the computer through RS-485 to realize system control and data collection. The data acquisition system is shown as Figure 4. The experimental device collects 64 characteristic parameters of the unit in real time, 48 of which are directly collected by the sensor, and the other 16 are calculated by the simulation software VisSim. The state data of each of the working conditions is collected at a sampling interval of 10s, forming the data set of the research presented in this paper.

### 2.3. Data Analysis

**Figure 5:**
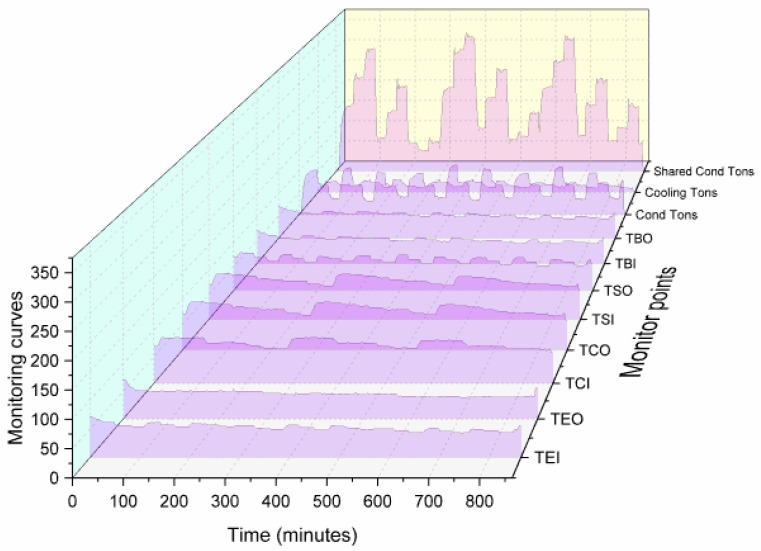
Visualization data.

In order to further explore the data characteristics, this section randomly selects 1-dimensional data for visualization, where TCO represents the temperature of condenser water out, TCI represents the temperature of condenser water in, Evap Tons represents the calculated 1st law energy balance for evaporator water, kW represents the compressor power, and RLA% represents the percent of maximum rated load amps. As shown in Figure 5, the collected state data form a typical time series and exhibit strong nonlinearity.

In this study, the data selection are as follows: firstly, the state parameters of the chiller when it starts will be similar to some fault conditions, so this study deletes the unsteady state data and only studies the steady state data; Secondly, remove irrelevant variables and strongly correlated variables, such as measurement time, because the measurement time has nothing to do with the fault classification of the unit. 

## 3. Model Preparation

In this section, the components of the proposed approach are introduced firstly, and then the architecture of the proposed 1D-CNN_GRU is described.

### 3.1. Long Short-Term Memory

Recurrent Neural Network (RNN) is a general term of neural network structure specially applied in processing sequence data that can handle the “long-term dependency” relationship of any time series. The problems of “gradient explosion” and “gradient dispersion” arise when “back-propagation” is applied to train a very deep RNN. Hochreiter and Schmidhuber, in 1997, first proposed Long Short Term Memory Neural Network (LSTM) to overcome the above issues [22]. 

The well-designed memory cells in the LSTM are applied instead of the activation function of the hidden state in RNN. There are three gates of input, forget, and output in the memory cells that are designed to preserve the previous information, update the cell state, and control the flow information. Each gate contains a sigmoid activation function in order to control whether they are triggered, conditionally changing the state. The forget gate uses the sigmoid activation function to decide the degree of forgetting the information from cell states. The output value of sigmoid is a real number between 0 and 1, where 1 means that all information is allowed to pass, and 0 implies that nothing is allowed to pass. Assuming a one-layer LSTM, the input gate, the forget gate, the output gate, the new cell memory, the updated cell state, and the output of the current hidden layer are calculated using Equations (1)–(6):

(1) $$i_{t}=\sigma \left(W_{i}x_{t}+W_{i}^{h}h_{t-1}+b_{i}\right)\;t=1,\;2,\;\cdots ,\;T$$

(2) $$f_{t}=\sigma \left(W_{f}x_{t}+W_{f}^{h}h_{t-1}+b_{f}\right)\;t=1,\;2,\;\cdots ,\;T$$

(3) $$o_{t}=\sigma \left(W_{o}x_{t}+W_{o}^{h}h_{t-1}+b_{o}\right)\;t=1,\;2,\;\cdots ,\;T$$

(4) $${\tilde{c}}_{t}=tanh\left(W_{c}x_{t}+W_{c}^{h}h_{t-1}+b_{c}\right)\;t=1,\;2,\;\cdots ,\;T$$

(5) $$c_{t}=c_{t-1}*f_{t}+{\tilde{c}}_{t}*i_{t}\;t=1,\;2,\;\cdots ,\;T$$

(6) $$h_{t}=o_{t}*tanh\left(c_{t}\right)\;t=1,\;2,\;\cdots ,\;T$$

where *W_i_*, *W_f_*, *W_o_*, and *W_c_* denote the feedforward weight matrices [23], and $W_{i}^{h}$, $W_{f}^{h}$, $W_{o}^{h}$, and $W_{c}^{h}$ denote the recurrent weight matrices, *b_i_*, *b_f_*, *b_o_*, and *b_c_* denote the bias vectors, the subscripts *i*, *f*, *o*, and *c* denote the input gate, forget gate, output gate, and cell unit, respectively, represents the sigmoid activation function [24].

### 3.2. Gated Recurrent Unit

In 2014, the gated recurrent unit (GRU) concept was suggested by Cho et al. [25] as an alternative type of gate-based recurrent unit in order to capture dependencies of different time scales adaptively, which is a variant of LSTM and has a smaller architecture with the LSTM unit. Compared to LSTM, GRU improves the training efficiency by simplifying connection and reducing trainable parameters on the premise of ensuring the memory ability of neurons. The GRU comprises the update gate and the reset gate; the former is combined with the forget gate and the input gate. Assuming a one-layer GRU, the update gate, the reset gate, the GRU output candidate, and the output of the current hidden layer are calculated as Equations (7)–(10):

(7) $$z_{t}=\sigma \left(W_{z}x_{t}+W_{z}^{h}h_{t-1}+b_{z}\right)\;t=1,\;2,\;\cdots ,\;T$$

(8) $$r_{t}=\sigma \left(W_{r}x_{t}+W_{r}^{h}h_{t-1}+b_{r}\right)\;t=1,\;2,\;\cdots ,\;T$$

(9) $${\tilde{h}}_{t}=tanh\left(W_{h}x_{t}+W_{h}^{h}\left(h_{t-1}*r_{t}\right)+b_{h}\right)\;t=1,\;2,\;\cdots ,\;T$$

(10) $$h_{t}=\left(1-z_{t}\right)*h_{t-1}+z_{t}*{\tilde{h}}_{t}\;t=1,\;2,\;\cdots ,\;T$$

where *W_z_*, *W_r_*, and *W_h_* denote the feedforward weight matrices, and $W_{z}^{h}$, $W_{r}^{h}$, and $W_{h}^{h}$ denote the recurrent weight matrices, *b_z_*, *b_r_*, and *b_h_* denote the bias vectors, the subscripts *z*, *r*, and *h* denote the update gate, reset gate, and hidden unit, respectively, σ represents the sigmoid activation function.

### 3.3. Convolution Neural Network

**Figure 6:**
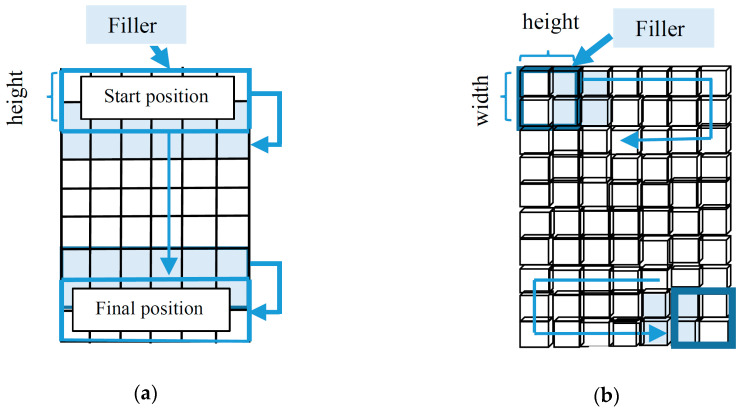
The sliding direction of the filter on 1D-CNN and 2D-CNN, respectively. (**a**) The sliding direction of the filter on 1D-CNN. (**b**) The sliding direction of the filter on 2D-CNN.

A typical feedforward neural network initially suggested by LeCun et al. [26] for image processing is convolutional neural networks (CNN). The main CNN features consist of shared weights and sparse connections. Shared weights, in CNN, may efficiently avoid overfitting of the algorithm, and sparse connection can reduce the number of training parameters [27]. The filter sliding direction on 1D-CNN and 2D-CNN are illustrated in Figure 6.

One-dimensional convolutional neural network (1D-CNN) is often applied to time-sequences, the convolutional output of which is one-dimensional. The computational details of 1D-CNN are shown as follows. Assume that the sequential input data is $X=[x_{1},x_{2},\;\ldots \;\ldots \;,x_{n}]\in ,R^{d\times n}$, where d and n denote the dimension and the length of the input sequence, respectively. The convolutional layers, seen as a collection of digital filters, convolve multiple local filters with raw input data and generate corresponding local features [28]. The specific convolution operation is given as follows:

(11) $$c_{i}=\varphi \left(w\cdot x_{i:i+m-1}+b\right)$$

Let $w\in ,R^{m\times d}$ signifies a filter vector; $x_{i:i+m-1}$ signifies an m-length sliding window starting from the i^th^ time step; · signifies the dot product; φ and b represent the non-linear activation function and bias, respectively; $c_{i}$ represents the activation of the filter w on the corresponding subsequence $x_{i:i+m-1}$. A feature map may be acquired by sliding the filtering window from the beginning time step to the ending time step [29]. Pooling layers aim to decrease the length of the feature map following several rules, including average and max.

### 3.4. Proposed Fault Diagnosis Approach

**Figure 7:**
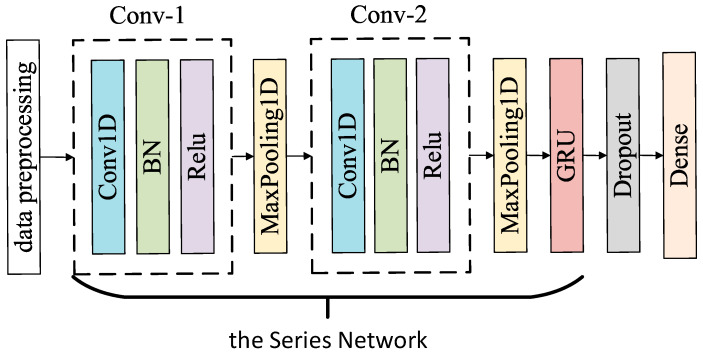
The proposed series network structure.

The basic idea of building a hybrid neural network of 1D-CNN and GRU is to connect two deep learning methods of 1D-CNN and GRU in series, in which 1D-CNN is the primary network of the series network, and GRU is the secondary network of the series network. The proposed series network structure is shown in Figure 7. In this study, GRU instead of LSTM is used as the secondary network of hybrid neural network, mainly to reduce the number of trainable parameters, memory demand, as well as training time. The comparison of LSTM with GRU trainable parameters is demonstrated in Table 3. The max-pooling layer is introduced in order to reduce the number of trainable parameters compared to the fully-connected layer. As demonstrated in Figure 7, the structure of 1D-CNN_GRU comprises the input, the hidden, and the output layers. The hidden layer consists of two convolution layers, 2 pooling layers, a GRU layer, as well as a fully connected layer [30]. 

**Figure 8:**
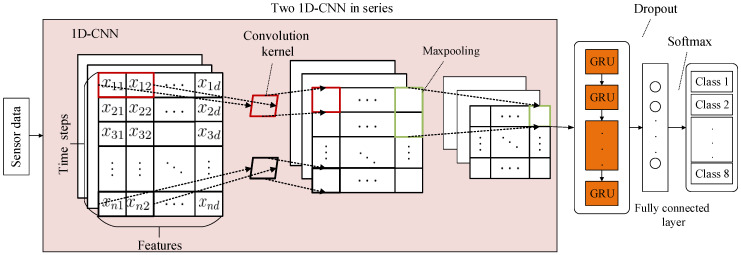
The Diagnosis Process of 1D-CNN_GRU.

Figure 8 illustrates the diagnosis process of 1D-CNN_GRU in detail. The approach comprises two essential steps: the first is adaptively extracting features from the collected multi-sensors data using 1D-CNN. The next is automatically learning the relation between different time steps in the features of 1D-CNN output [30]. In addition, the proposed approach preserves the speed of 1D-CNN and the order-sensitivity of GRU, simultaneously.

In this research, the rectified linear unit (ReLU) [31] was chosen as the activation function of the hidden layer. Compared with other activation functions, such as sigmoid, tanh, ReLU has the following advantages: (1) For linear functions, ReLU has stronger expression ability; (2) For nonlinear functions, the gradient of ReLU in the non-negative range is constant, so there is no vanishing gradient problem, which keeps the convergence rate of the model in a stable state. ReLU expression is shown as Equation (12):

(12) $$ReLU\left(x\right)=\left\{ \begin{array}{l} x\;if\;x>0\\ 0\;if\;x\leq 0 \end{array}\right.$$

**Figure 9:**
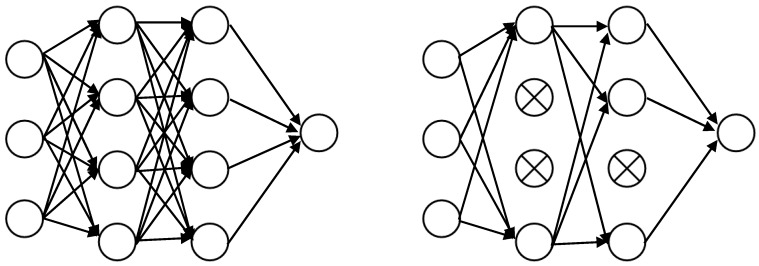
The technical diagram of dropout.

In addition, dropout was introduced to address overfitting [32,33]. In the actual network model training process, dropout will set some hidden layer neurons to 0, and then these neurons will not play a role in the forward propagation process of network training. Dropout technical diagram is shown in Figure 9.

In the dense layer, softmax is chosen as a classifier to classify GRU output. Suppose there are *m* labeled training set, $\left\{\left(x^{\left(1\right)},y^{\left(1\right)}\right),\cdots ,\left(x^{\left(m\right)},y^{\left(m\right)}\right)\right\}$, $y_{i}\in \left\{1,2,\cdots ,k\right\}$, *k* denotes the number of label. For a given input sample *x*, the probability  p(y=j|x) of each sample belonging to each class *j* is estimated by a hypothesis function. The hypothesis function outputs a k-dimensional vector to represent the k estimated probability values [34]. The hypothesis function is shown as Equation (13):

(13) $$h_{\theta }\left(x^{\left(i\right)}\right)=\left[\begin{array}{c} p\left(y^{\left(i\right)}=1|x^{\left(i\right)};\theta \right)\\ p\left(y^{\left(i\right)}=2|x^{\left(i\right)};\theta \right)\\ M\\ p\left(y^{\left(i\right)}=k|x^{\left(i\right)};\theta \right) \end{array}\right]=\frac{1}{\sum_{j=1}^{k}e^{\theta_{j}^{T}x^{\left(i\right)}}}\left[\begin{array}{c} e^{\theta_{1}^{T}x^{\left(i\right)}}\\ e^{\theta_{2}^{T}x^{\left(i\right)}}\\ M\\ e^{\theta_{k}^{T}x^{\left(i\right)}} \end{array}\right]$$

where $\theta_{1},\theta_{2},\cdots ,\theta_{k}\in {ℜ}^{n+1}$ denote the model parameter. $\overset{k}{\underset{j=1}{\sum }}e^{\theta_{j}^{T}x^{\left(i\right)}}$ stands for normalizing the probability distribution so that the sum of all probabilities is 1. Therefore, in softmax regression, the probability of dividing sample *x* into label *j* is shown in Equation (14):

(14) $$p\left(y^{\left(i\right)}=j|x^{\left(i\right)};\theta \right)=\frac{e^{\theta_{j}^{T}x^{\left(i\right)}}}{\sum_{l=1}^{k}e^{\theta_{l}^{T}x^{\left(i\right)}}}$$

It is typically better to use cross-entropy error than mean squared error to assess the quality of the network in a multi-classification investigation. Therefore, this study adopted the multi-class cross entropy function as the loss function of the model. The categorical cross-entropy functions for the binary classes is well-defined as:

(15) $$loss=-\frac{1}{m}\sum_{j=1}^{m}\sum_{i=1}^{n}y_{ji}log{\hat{y}}_{ji}$$

where *m* denotes the number of samples, *n* denotes the class, $y_{ji}$ represents the true probability for the $i^{th}$ class, and ${\hat{y}}_{ji}$ represents the predicted probability.

## 4. Experimental Process and Analysis

### 4.1. Diagnosis Process

ASHRAE RP-1043 is very appropriate for fault diagnosis study due to the following reasons: Firstly, a wide variety of chiller faults is studied; secondly, the faults are introduced at four levels of severity; thirdly, a complete set of sensor data is collected from the chiller at a sampling rate of 10 seconds.

**Figure 10:**
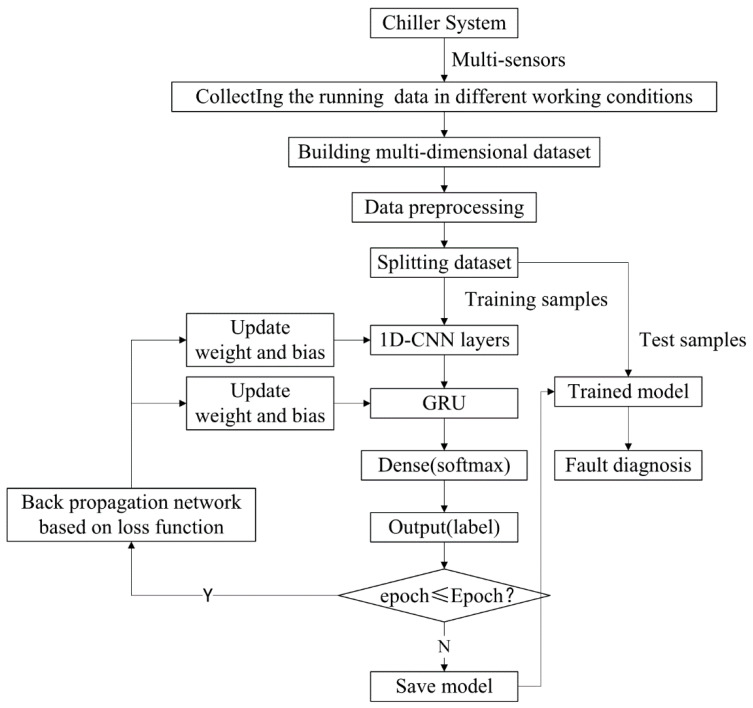
The flowchart of feature extraction and fault diagnosis based on 1D-CNN_GRU.

The idea of 1D-CNN_GRU hybrid neural network fault diagnosis is to scramble the sample data as the input of 1D-CNN network model, and use the convolutional layer and pooling layer of 1D-CNN to extract the local features of the samples, determine the optimal parameters of the 1D-CNN network model and apply the parameters to the primary network of the hybrid neural network. Subsequently, the sequence data without scrambled order is used as the input of the hybrid network model. In the process of feature extraction, the local features of the sequence are extracted by 1D-CNN firstly, then the output of 1D-CNN is used as the input of GRU to further extract the long-term dependent features of the sequence, and finally achieve accurate fault diagnosis. The specific diagnosis process is shown in Figure 10:

- Step 1: Collecting data samples of different working conditions by sensors and building multi-dimensional fault samples;
- Step 2: Normalizing samples by z-scores. There are differences in the units of different attribute values, which have a particular impact on the convergence speed and precision of the model. z-scores are computed applying the mean and standard deviation along each column for the collected sample matrix. The normalized data value $x^{*}$ is calculated as:
  (16) $$x^{*}=\frac{x_{i}-\bar{x}}{s}$$
  where $x_{i}$ denotes one column of the original sample matrix, $\bar{x}=\frac{1}{n}\sum_{i=1}^{n}x_{i}$ denotes the average of $x_{i}$, $s=\sqrt{\frac{1}{n-1}\sum_{i=1}^{n}{\left(x_{i}-\bar{x}\right)}^{2}}$ denotes the standard deviation of $x_{i}$, and n denotes the number of samples;
- Step 3: Splitting the dataset into training, validation, and test samples. This paper randomly picks up 70 percent of samples as the training dataset and 10 percent as the validation samples; the remaining samples are used as the test dataset to assess the performance of the diagnosis model;
- Step 4: Training the hybrid algorithm with training samples. In the training process, batch normalization is chosen to accelerate the training of the network [30], dropout is introduced to address the problem of overfitting, and back-propagation network is used to update weights and biases;
- Step 5: Taking the faults with four levels of severity as the input of the model, respectively, to verify the effectiveness and sensitivity of the algorithm. The performance of the diagnostic model is assessed based on the predicted and true labels.

### 4.2. Evaluation Index

The index to evaluate the diagnosis effect is generally the accuracy, which is defined as the proportion of correctly diagnosed samples to the total samples for a given sample. However, this indicator does not perform well when the positive and negative samples are unbalanced. For example, there are 9900 positive samples and 100 negative samples. If the model predicts all positive samples to be positive, the accuracy rate is 99%. Although the accuracy is high, it is not persuasive to use this indicator alone, because it can not fully compare the advantages and disadvantages of the model. Therefore, this study uses multiple evaluation indicators to comprehensively reflect the performance of the model, including Accuracy, Precision, Recall, and F1-measure, they are calculated using Equations (17)–(20):

(17) $$Accuracy=\frac{\left|TP\right|+\left|TN\right|}{\left|TP\right|+\left|FP\right|+\left|TN\right|+\left|FN\right|}$$

(18) $$Precision=\frac{\left|TP\right|}{\left|TP\right|+\left|FP\right|}$$

(19) $$Recall=\frac{\left|TP\right|}{\left|TP\right|+\left|FN\right|}$$

(20) $$F1-measure=2*\frac{Precision*Recall}{Precision+Recall}$$

where TP, FP, TN, and FN denote the true-positive, false-positive, true-negative and false- negative, respectively. True-positive represents the number of positive samples correctly diagnosed, false-positive represents the number of negative samples incorrectly diagnosed, true-negative represents the number of negative samples correctly diagnosed, false-negative represents the number of positive samples incorrectly diagnosed.

### 4.3. Parameter Optimization

The grid search method of scikit-learn was introduced to optimize network parameters and the hyper-parameters in this paper. The experimental results are shown in Table 4. Supposing the number of filters in convolution layer is named NFC, the convolution kernel size in convolution layer is named KSC, the pooling size in maxpooling layer is named PSM, and the number of neurons in GRU is named NNG. In this experiment, the batch size and the epoch were set to 200 and 15, respectively, and randomly selected 4000 sample data for training.

As can be seen from Table 4 the performance of M9 is better than that of the others, the accuracy is 91%, the loss is 0.24, and the training speed is fast. The number of filters in convolution layer is 128, the convolution kernel size in convolution layer is 6, the pooling size in maxpooling layer is 2, and the number of neurons in GRU is 256, respectively.

**Figure 11:**
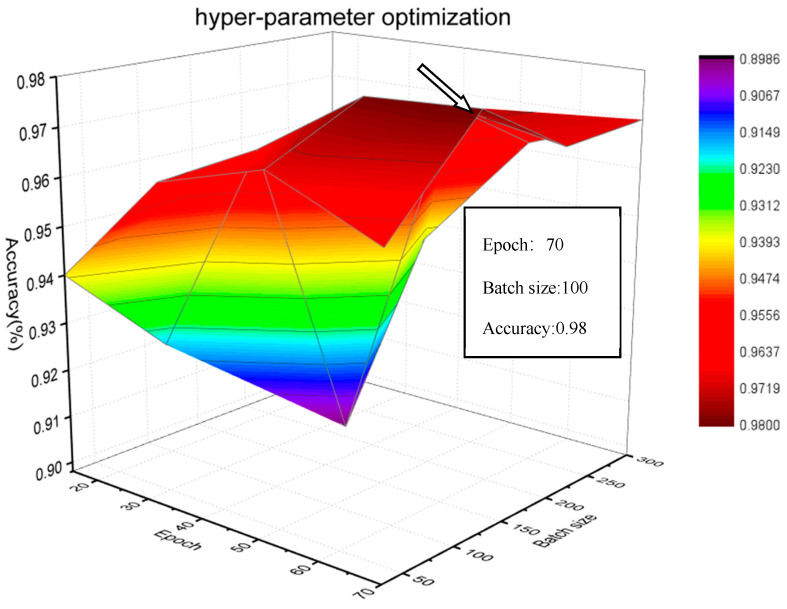
Relationship between accuracy and two key parameters.

Epoch and batch size are two very important parameters in fault diagnosis, which jointly determine the feature space of different working conditions, and then affect the final diagnosis results. In this experience, the number of filters in convolution layer, the convolution kernel size in convolution layer, the pooling size in maxpooling layer, and the number of neurons in GRU are set to 128, 6, 2 and 256, respectively. It can be seen from Figure 11, the performance of model is superior to other models when epoch and batch size are 70 and 100, respectively.

### 4.4. Sensitivity Evaluation

About 160,000 samples were extracted from the experimental database with the severity increasing from level 1 to level 4. During the experiment, the training set, validation set and test set were divided according to the ratio of 8:1:1. The parameters used in fault diagnosis is shown in Table 5.

**Figure 12:**
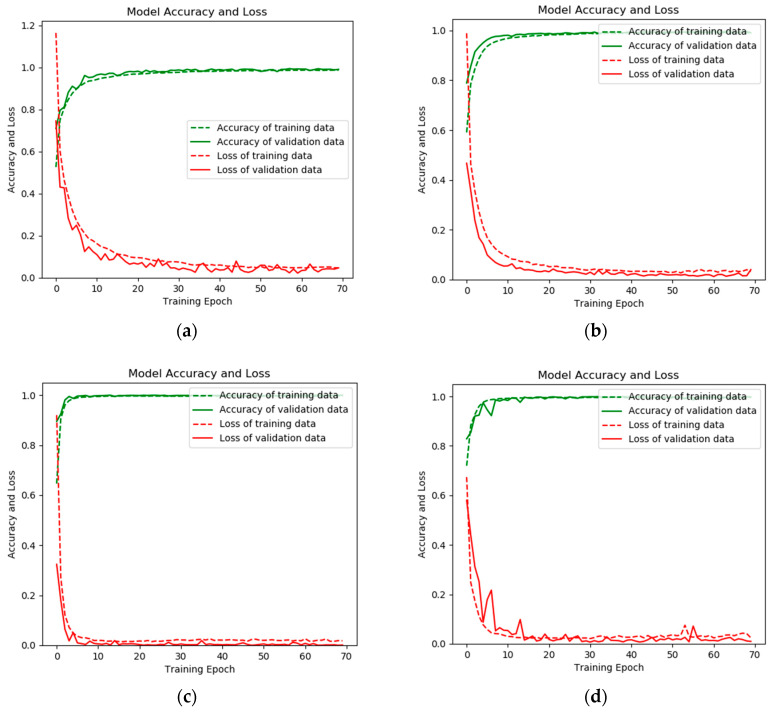
The loss value and accuracy of both the training and validation processed on RP-1043 with fault severity (**a**) level I, (**b**) level II, (**c**) level III, and (**d**) level IV.

As can be seen in Figure 12, the red dotted curve represents the loss of training data, the red solid curve represents the loss of validation data, the green dotted curve represents the accuracy of training data, the green solid curve represents the accuracy of validation data. The convergence speed is very fast, and there is no overfitting phenomenon during the training and validation process no matter how serious the fault.

**Figure 13:**
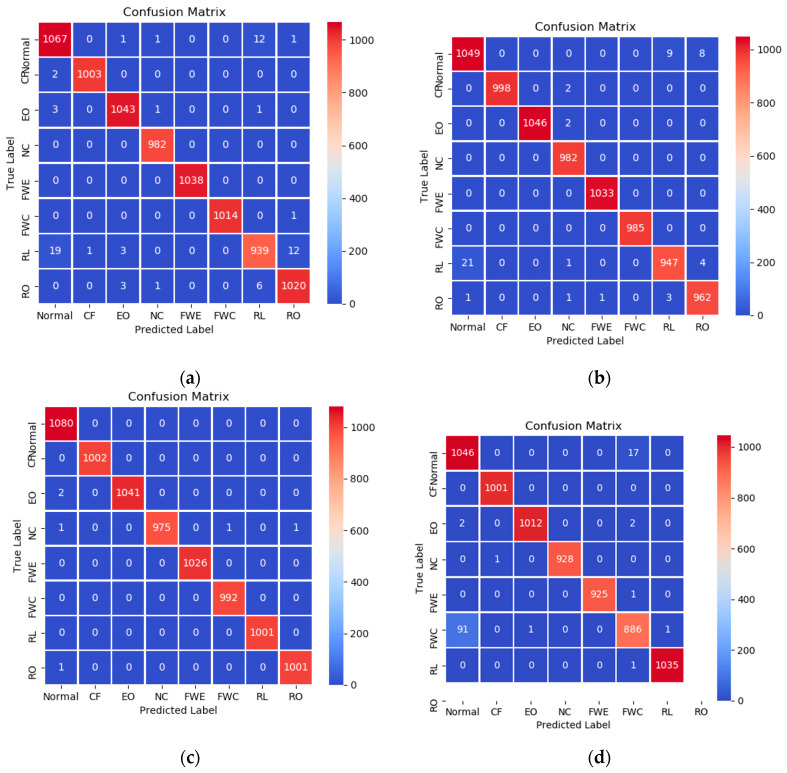
The confusion matrix with different fault level with fault severity (**a**) level I, (**b**) level II, (**c**) level III, and (**d**) level IV.

A confusion matrix is an effective visualization tool for the performance of a classification approach. Each row in the confusion matrix signifies the true label, while each column signifies the predicted label [35]. The confusion matrix recorded the testing classification results as shown in Figure 13. Among the test dataset with fault degree 1, the diagnosis accuracy of the normal case (Normal), condenser fouling (CF), excess oil (EO), non-condensable refrigerant (NC), reduced evaporator water flow (FWE), reduced condenser water flow (FWC), refrigerant leak (RL), and refrigerant overcharge (RO) can be as seen in Figure 13a; they are 98.6%, 99.8%, 99.5%, 100%, 100%, 99.9%, 96.4%, and 99%, respectively. Simultaneously, Figure 13b–d also indicate that 1D-CNN_GRU has excellent performance on test data with fault degree 2–4. The least diagnosis rate is 97.3% in the experiments on test dataset with fault degree 2, while the least diagnosis rate are 94.3% and 90.5% in the experiments on test dataset with fault degree 3 and fault degree 4. 

The precision of the model and the recall affect each other. There will be some problems such as missed alarms, false alarms and false alarms during the operation of the cold machine system. When there is a large amount of missed alarms, the model tends to give fewer alarms. In this case, although the real fault is diagnosed, some faults are still not identified, resulting in a low precision rate and a high recall rate. On the contrary, when the number of false alarms or false alarms during operation is large, the model tends to have more alarms, and then a correct sample can be diagnosed as a fault, resulting in higher accuracy and lower recall rate, but the precision and recall of the better models should be very high at the same time. Therefore, the comprehensive evaluation index (F1-measure) is proposed as a balance point between the two to integrate the recall rate and accuracy index. 

It can be seen from Table 6 that the precision and recall of the proposed diagnosis model are close to 1 for each type of fault at each fault severity. In addition, the comprehensive evaluation index of the comprehensive precision rate and recall is also close to 1. Therefore, the proposed approach is sensitive to the type and severity of fault.

### 4.5. Effectiveness Evaluation

**Figure 14:**
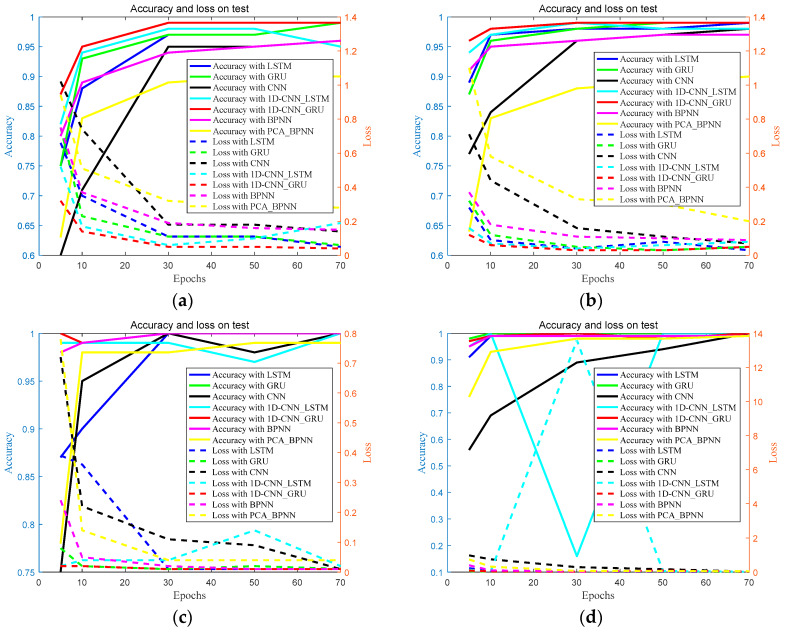
Comparison experimental results with fault severity (**a**) level I, (**b**) level II, (**c**) level III, and (**d**) level IV.

In order to evaluate the effectiveness of the proposed approach, comparative experiments were conducted on the same dataset, including GRU, LSTM, 1D-CNN, BPNN, PCA_BPNN, as well as 1D-CNN_LSTM. Based on these experience results, shown in Figure 14, it can be concluded that:

(1) The experimental results showed that the accuracy of deep learning is obviously higher than that of traditional neural network approaches. It can be explained that deep learning can adaptively learn the valuable information from the raw sensor data, and the proposed method can combine the speed of 1D-CNN with the order-sensitivity of GRU.
(2) As can be seen from Figure 14, the lighter the fault severity, the better the performance of the proposed approach. It can be revealed that the proposed fault diagnosis approach has a reasonable identification rate for minor faults.

## 5. Conclusions

In order to improve the performance of water chiller fault diagnosis and sensitivity to different fault degrees, a hybrid approach based on 1D-CNN and GRU is proposed in this paper, which combines the advantages of 1D-CNN and GRU. Firstly, the parameters of the proposed approach are optimized by experiments, and then the sensitivity and effectiveness of the proposed approach are verified by experiments. The experimental results show that: (1) The proposed approach is more sensitive to micro faults; (2) The proposed approach can adaptively extract the features of different faults and achieve high accuracy when the number of iterations is very small; (3) The proposed approach has a good performance for different fault severity. Therefore, the model provides support for the daily maintenance of the refrigeration system of IDC, and ensures the normal operation of the network.

## 6. Future Works

The fault diagnosis approach based on deep learning also has many difficulties to overcome. Firstly, the fault diagnosis of deep learning approach is limited to the existing fault types and deep learning has no ability to identify new faults. Secondly, there is no systematic deep learning tuning theory knowledge. The tuning of model parameters needs to be based on experience. Thirdly, fault diagnosis requires the system to be able to identify the type of fault and determine the location of the fault in a timely and fast manner, but the training of deep learning models is time consuming.

In the current fault diagnosis research, labeled data feature learning plays an important role. However, in the practical application of fault diagnosis, the occurrence of fault data is often unlabeled, and the quantity of fault data is huge. Labeling fault data is a VERY tedious thing. Therefore, in the future, the unlabeled data feature learning and the diagnosis technology will be paid more and more attention.

## Figures and Tables

**Table 1:** Summary of chiller faults.

Health Condition	Description of Fault	Label
Healthy	Normal	Normal
Faulty	Condenser Fouling	CF
	Excess Oil	EO
	Non-condensable in Refrigerant	NC
	Reduced Evaporator Water Flow	FWE
	Reduced Condenser Water Flow	FWC
	Refrigerant Leak	RL
	Refrigerant Overcharge	RO

**Table 2:** Fault Level Classification.

Category	Normal	Level 1	Level 2	Level 3	Level 4
CF	164 tubes	−12%	−20%	−30%	−45%
EO	22 lbs	−14%	−32%	−50%	−68%
NC	no nitrogen	+1%	+2%	+3%	+5%
FWE	216 gmp	−10%	−20%	−30%	−40%
FWC	270 gmp	−10%	−20%	−30%	−40%
RL	300 lbs	−10%	−20%	−30%	−40%
RO	300 lbs	+10%	+20%	+30%	+40%

**Table 3:** The Comparison of LSTM with GRU Trainable Parameters.

Comparison	LSTM	GRU
Number of gates	3	2
Number of weight matrices	8	6
Number of bias vectors	4	3
Number of matrix multiplies	8	6

**Table 4:** Parameters listed with the training time, loss, and accuracy of model performance.

Model	NFC	KSC	PSM	NNG	Acc	Loss	Time
M1	128	3	2	64	0.81	0.47	54.5 s
M2	128	3	2	128	0.88	0.34	80.3
M3	128	3	2	256	0.89	0.3	104.4
M4	128	3	3	64	0.79	0.57	35.5
M5	128	3	3	128	0.82	0.5	39.4
M6	128	3	3	256	0.84	0.43	51
M7	128	6	2	64	0.88	0.33	72.7
M8	128	6	2	128	0.87	0.32	72.2
M9	128	6	2	256	0.91	0.24	98.2
M10	128	6	3	64	086	0.39	42.2
M11	128	6	3	128	0.89	0.35	44.2
M12	128	6	3	256	0.88	035	60.2
M13	128	9	2	64	0.88	0.3	65.4
M14	128	9	2	128	0.88	0.33	70.8
M15	128	9	2	256	0.88	0.35	96
M16	128	9	3	64	0.87	0.35	38.9
M17	128	9	3	128	0.90	0.28	45.3
M18	128	9	3	256	0.90	0.29	51.8
M19	256	3	2	64	0.81	0.46	140.1
M20	256	3	2	128	0.87	0.33	152.1
M21	256	3	2	256	0.9	0.25	180.3
M22	256	3	3	64	0.79	0.55	82.2
M23	256	3	3	128	0.83	0.46	82.8
M24	256	3	3	256	0.85	0.46	107.4
M25	256	6	2	64	0.88	0.32	222.5
M26	256	6	2	128	0.90	0.26	226.9
M27	256	6	2	256	0.90	0.27	307.8
M28	256	6	3	64	0.86	0.37	184.7
M29	256	6	3	128	0.84	0.43	179.6
M30	256	6	3	256	0.85	0.47	177.1
M31	256	9	2	64	0.89	0.32	267.4
M32	256	9	2	128	0.88	0.33	300.9
M33	256	9	2	256	0.9	0.29	279.8
M34	256	9	3	64	0.81	0.44	214.5
M35	256	9	3	128	0.85	0.45	194.4
M36	256	9	3	256	0.9	0.29	207.7

**Table 5:** The parameters used in fault diagnosis.

Description	Value
the number of filters in convolution layer	128
the convolution kernel size in convolution layer	6
the pooling size in maxpooling layer	2
the number of neurons in GRU	256
the number of neurons in dense	8
dropout	0.2
batch size	100
epoch	100

**Table 6:** Classification report.

Fault Level	Label	Description	Precision	Recall	F1-Measure	Support
I	0	Normal	0.98	0.99	0.98	1082
I	1	CF	1.00	1.00	1.00	1005
I	2	EO	0.99	1.00	0.99	1048
I	3	NC	1.00	1.00	1.00	982
I	4	FWE	1.00	1.00	1.00	1038
I	5	FWC	1.00	1.00	1.00	1015
I	6	RL	0.98	0.96	0.97	974
I	7	RO	0.99	0.99	0.99	1030
II	0	Normal	0.98	0.98	0.98	1066
II	1	CF	1.00	1.00	1.00	1000
II	2	EO	1.00	1.00	0.99	1048
II	3	NC	0.99	1.00	1.00	982
II	4	FWE	1.00	1.00	1.00	1033
II	5	FWC	1.00	1.00	1.00	985
II	6	RL	0.99	0.97	0.98	973
II	7	RO	0.99	0.99	0.99	968
III	0	Normal	1.00	1.00	1.00	1080
III	1	CF	1.00	1.00	1.00	1002
III	2	EO	1.00	1.00	1.00	1043
III	3	NC	1.00	1.00	1.00	978
III	4	FWE	1.00	1.00	1.00	1026
III	5	FWC	1.00	1.00	1.00	992
III	6	RL	1.00	1.00	1.00	1001
III	7	RO	1.00	1.00	1.00	1002
IV	0	Normal	0.92	0.98	0.95	1063
IV	1	CF	1.00	1.00	1.00	1001
IV	2	EO	1.00	1.00	1.00	1016
IV	3	NC	1.00	1.00	1.00	929
IV	4	FWE	1.00	1.00	1.00	126
IV	5	FWC	1.00	1.00	1.00	992
IV	6	RL	0.98	0.91	0.94	979
IV	7	RO	1.00	1.00	1.00	1036
